# High-Performance Optical Fiber Displacement Sensor with Extended Linear Range and Sensitivity

**DOI:** 10.3390/s25020418

**Published:** 2025-01-12

**Authors:** Gorka Zubia, Joseba Zubia, Josu Amorebieta, Gotzon Aldabaldetreku, Asier Zubia, Gaizka Durana

**Affiliations:** 1 Department of Graphical Expression and Project Engineering, University of the Basque Country, 48013 Bilbao, Spain; 2 CREOL, The College of Optics and Photonics, University of Central Florida, Orlando, FL 32816, USA; 3 Department of Communications Engineering, University of the Basque Country, 48013 Bilbao, Spain; joseba.zubia@ehu.eus (J.Z.);; 4 EHU Quantum Center, University of the Basque Country, 48013 Bilbao, Spain; 5 Department of Applied Mathematics, University of the Basque Country, 48013 Bilbao, Spain; 6 TECNALIA, Basque Research and Technology Alliance, 48160 Derio, Spain

**Keywords:** optical fiber sensor, intensity-modulated optical sensor, optical fiber bundle design, displacement sensor, structural health monitoring, aeronautical sensing, tip timing, tip clearance

## Abstract

Optical Fiber Displacement Sensors (OFDSs) provide several advantages over conventional sensors, including their compact size, flexibility, and immunity to electromagnetic interference. These features make OFDSs ideal for use in confined spaces, such as turbines, where direct laser access is impossible. A critical aspect of OFDS performance is the geometry of the fiber bundle, which influences key parameters such as sensitivity, range, and dead zones. In this work, we present a streamlined design methodology for azimuthally symmetric OFDSs to improve the linear range of these sensors. The most effective configuration we propose is the pentafurcated bundle, which consists of a central transmitting fiber surrounded by four concentric rings of fibers with different radii. Our experimental results show that the pentafurcated designs increase both the range—up to 10.5 mm—and the sensitivity of the sensor—$2{\mathrm{mm}}^{-1}$—while minimizing the dead zone of the sensor (2.5 mm), allowing accurate measurements even at very short distances

## 1. Introduction

Accurate measurement of displacement is fundamental in Industry 4.0, where advanced sensing technologies enable structural health monitoring and the optimization of industrial processes. Displacement sensors play a crucial role across diverse applications, including the measurement of surface deformation, bending, material thickness, height, roughness, and distances between components in turbines [1,2].

The aerospace industry, driven by the European Green Deal’s vision of zero-emission, demands innovative solutions for enhanced engine efficiency. New-generation aircraft engines, such as the UltraFan, demonstrate a 75% reduction in carbon emissions, 40% lower ${\mathrm{NO}}_{x}$ emissions, a 35% noise reduction, and 25% improved fuel efficiency compared to earlier Trent engines [3,4]. These improvements necessitate monitoring critical structural health parameters, such as tip clearance (TC) and tip timing (TT), with high-resolution sensors.

TC is the gap between a turbine or compressor blade tip and its casing, and is often less than 5 mm [5]. TC is essential to maximize efficiency by minimizing air and gas leakage, while preventing blade–casing contact [6]. TT tracks the arrival time of blades to assess axial, radial, and tangential vibrations, which are related to blade damage [7].

Traditional sensing methods—capacitive, inductive, and microwave—struggle to meet the rigorous demands of these applications. Fiber optical sensors are particularly well-suited for these tough environments due to their electromagnetic immunity and small size, and cover a variety of technologies and applications [8].

A wide range of fiber types is used in sensing applications, from basic single-mode fibers to more advanced solutions such as multicore fibers (MCFs) [9,10] and anti-resonant hollow-core fibers (ARHCFs) [11,12,13]. Additionally, researchers have proposed microstructured optical fibers (MOFs)—made of glass and POF—which offer enhanced sensitivity in gas, strain, or displacement sensing [14,15]. Functionalized fibers using specialized coatings have also proven useful for biochemical, temperature, or pressure sensing, often providing improved selectivity and higher signal-to-noise ratios [16]. Furthermore, SMS (single-mode–multimode–single-mode) fiber geometries present a compact and robust platform for interference-based or intensity-modulated sensors, enabling high resolution in displacement or refractive index measurements [17,18]. While these specialty fibers demonstrate strong potential at the laboratory scale, their commercial availability, higher fabrication costs, and sometimes complex interrogation schemes can challenge widespread deployment—particularly in harsh environments like aircraft engines. By contrast, intensity-modulated OFDSs remain one of the simplest and most cost-effective solutions, relying on the modulation of reflected light intensity to measure displacement [19,20]. Despite their promise, existing OFDS designs often lack versatility and require tailored configurations for different engine stages, motivating the work presented in this paper.

This work addresses these limitations by introducing a refined design methodology for OFDSs. We propose and experimentally validate two novel configurations, tetrafurcated and pentafurcated bundles, which significantly enhance the working range (up to 10.5 mm) and sensitivity (2 ${\mathrm{mm}}^{-1}$) while minimizing dead zones. These designs pave the way for versatile OFDSs to meet diverse industrial and aeronautical sensing needs [21].

## 2. Working Principle of the Optical Fiber Displacement Sensor

**Figure 1:**
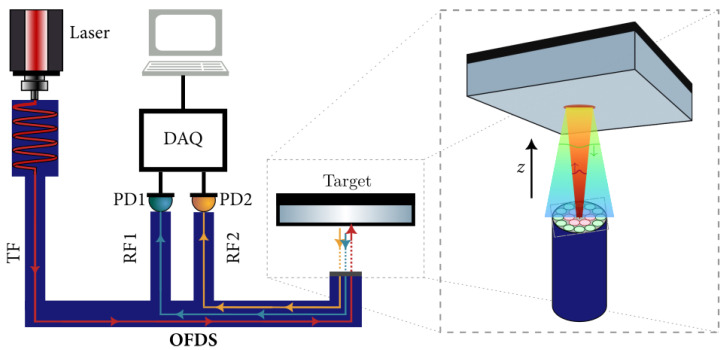
Typical OFDS experimental set-up. From **left** to **right**, a laser source coupled to the TF, the bundle, a computer, an acquisition card, the photodetectors—one per RF collection—and the target.

The typical experimental set-up of an OFDS is shown in Figure 1. It is composed of a fiber bundle, one photodetector per receiving fiber (RF) collection, an acquisition card, a mirror, and a laser light source [22,23].

To measure the OFDS’s response η(z) at various distances *z*, we place a target—a mirror or blade tip—on a motorized linear stage directly in front of the bundle tip. By incrementally changing the target position in this stage, we vary *z*. At each *z* position, the photodetectors measure the voltage signals $\left\{V_{1},V_{2},\ldots \right\}$ corresponding to the optical power collected by each RF collection. These voltages are recorded and processed by the acquisition card (DAQ).

**Figure 2:**
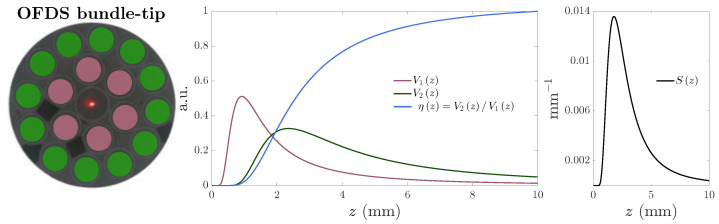
(**Left**): Trifurcated OFDS bundle tip with the single-mode TF at the center, and the 1st and 2nd RF collections in red and green. (**Center**): OFDS responsivity η(z) (blue) and voltages from optical powers collected by the 1st and 2nd RF collections (red/green). (**Right**): OFDS sensitivity S(z).

It has already been demonstrated that the geometrical configuration of the fiber bundle which provides a good trade-off between the range and sensitivity of the measurements, are the trifurcated OFDSs [24,25,26]. These have a single mode TF at their center and two collection of multimode RF as depicted in Figure 2a. Figure 2b presents the characteristic curve measured by the first ($V_{1}$, red) and second ($V_{2}$, green) RF collections of the OFDS, as well as the complete sensor response, named responsivity. The responsivity, $\eta \left(z\right)$, and its sensitivity, $S\left(z\right)$, are given by [27],

(1) $$\eta \left(z\right)=\frac{V_{2}}{V_{1}}\;\mathrm{and}\;S\left(z\right)=\frac{d\eta \left(z\right)}{dz}.$$

We used a differential definition of responsivity to eliminate all measurement dependencies unrelated to distance, such as laser fluctuations, fiber bending, the reflectivity of the target surface, fiber attenuation, etc. The OFDS’s sensitivity $S\left(z\right)$ is defined as the derivative of the responsivity with distance. The maximum sensitivity is achieved, indeed, within the working range, as shown in Figure 2c.

**Figure 3:**
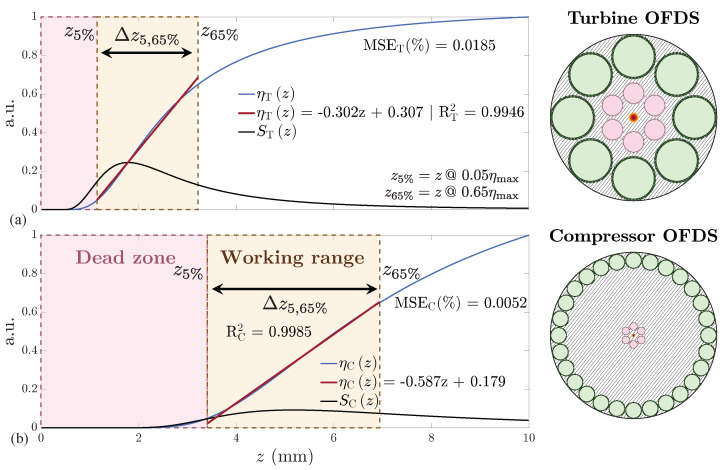
Typical responsivity $\eta \left(z\right)$ of two trifurcated OFDSs developed for a turbine and compressor stages of an aircraft engine. The yellow range represents its working range $\Delta z_{5,65}$, the red range represents the dead zone, and the brown line represents the estimated regression. The top panel shows the responsivity of an OFDS designed for TC measurements in the turbine of an aircraft engine (**a**) and its corresponding bundle tip. The red fibers are the 1st RF collection, and the greens, the 2nd. At the bottom, the OFDS for the compressor stage is shown (**b**). The working ranges were fixed between [1.5, 3] and [5, 9] mm.

The linear or working range of the OFDS contains the responsivity values regressed for TC calculation, and is shown in brown in Figure 3a. We define the working range as the distance range between the 5% ($z_{5}$) and the 65% ($z_{65}$) of the maximum responsivity $\eta_{\max}$; we denote it with $\Delta z_{5,65}$. The Appendix A shows that this corresponds to a Pearson’s correlation coefficient over 0.997 [28]. We refer to the dead zone of the OFDS as the range where the responsivity is too small, usually less than 5% of $\eta_{\max}$.

The major drawback of these trifurcated OFDSs is their lack of flexibility since they are designed ad hoc for each specific application; i.e., for the turbine or compressor stages, it is necessary to use different OFDSs. Figure 3a,b demonstrate that even measuring at different stages of the same engine, such as the turbine and compressor, two OFDSs with different geometrical configurations must be designed and manufactured. This is expensive and incurs into manufacturing delays. Therefore, the aeronautical industry needs to develop distance sensors with a wider linear range to be valid for all stages of the engines. That, while maintaining a high sensitivity, constitutes the main objective of this work.

## 3. Theoretical Model

### 3.1. Toy Model Approach

In this section, we present the theoretical framework used to design and analyze our tetra- and pentafurcated OFDSs. While the fundamental Gaussian beam and ring-based concepts have been described in earlier works [27,29], here we extend those methods to multi-ring arrangements with additional RF collections. This adaptation enables a significantly wider linear range without compromising sensor sensitivity.

**Figure 4:**
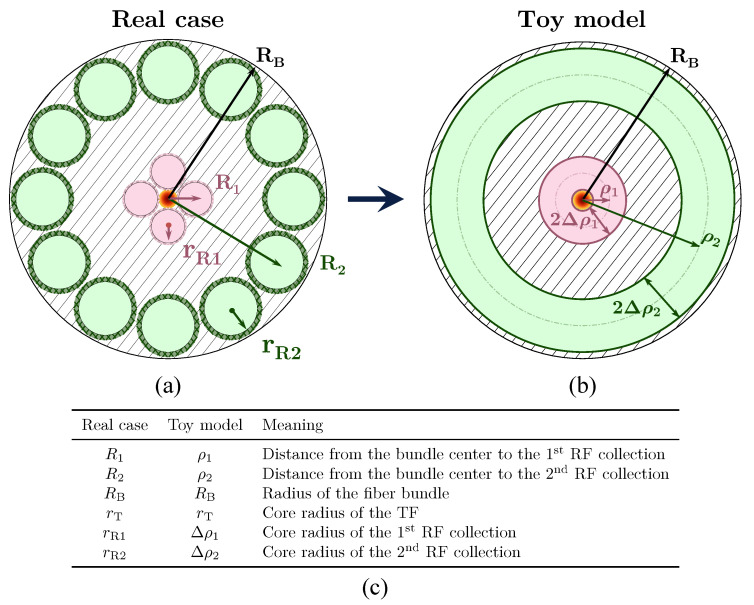
The real (**a**) and toy model (**b**) bundle tips: the red ring emulates the 1st RF collection, the green ring emulates the 2nd. (**c**) The cross-section equivalences between OFDS bundle tips.

We begin with a toy model approach that replaces the discrete RF arrangement by continuous receiving rings, see Figure 4, simplifying the calculation of the optical power collected by each ring. The original formulations, described in [27,29], capture the essential physics—beam divergence, target reflectivity, etc.—while greatly reducing computational complexity. In this work, we propose an extension of that approach to accommodate additional receiving rings—e.g., tetra- and pentafurcated bundles—as shown in Equation (2) and further detailed in Section 3.4.

The toy model approach was chosen to simplify the analysis of OFDS configurations. By approximating discrete fibers as continuous rings, the model significantly reduces computational complexity while preserving essential geometric and optical characteristics. This abstraction enables quick evaluation of critical design parameters, such as working range, sensitivity, and linearity, providing actionable insights for sensor optimization. The toy model serves as an efficient alternative to computationally intensive simulations, maintaining strong agreement with experimental results [29].

**Figure 5:**
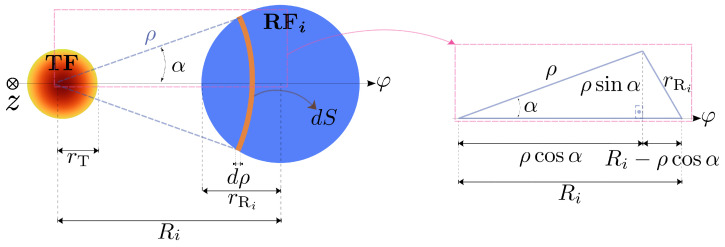
Geometry breakdown for calculating power gathered by each RF collection, with ρ and *z* as the radial and longitudinal coordinates.

The responsivity of the OFDS is calculated using the Gaussian beam approach [30,31] using Equation (2), with *k* representing the tunable gain quotient $G_{2}/G_{1}$. Figure 5 illustrates the geometry.

(2) $$\eta \left(z\right)=\frac{V_{2}}{V_{1}}=k\frac{\int_{R_{2}-r_{R_{2}}}^{R_{2}+r_{R_{2}}}\exp\;\left(\;-\frac{2\rho^{2}}{w^{2}\left(z\right)}\right)\cos^{-1}\left(\frac{1}{2}\left(\frac{\rho }{R_{2}}+\frac{R_{2}}{\rho }-\frac{r_{R_{2}}^{2}}{\rho R_{2}}\right)\right)\rho d\rho }{\int_{R_{1}-r_{R_{1}}}^{R_{1}+r_{R_{1}}}\exp\;\left(\;-\frac{2\rho^{2}}{w^{2}\left(z\right)}\right)\cos^{-1}\left(\frac{1}{2}\left(\frac{\rho }{R_{1}}+\frac{R_{1}}{\rho }-\frac{r_{R_{1}}^{2}}{\rho R_{1}}\right)\right)\rho d\rho }\;$$

Thanks to the toy model, Equation (2) simplifies to [27]:

(3) $$\eta \left(z\right)=p\exp\left(-\frac{q}{z^{2}}\right)$$

where parameters *p* and *q* are given by

(4) $$p=\frac{\rho_{2}\Delta \rho_{2}}{\rho_{1}\Delta \rho_{1}}\;\mathrm{and}\;q=\frac{\rho_{2}^{2}+\Delta \rho_{2}^{2}-\rho_{1}^{2}-\Delta \rho_{1}^{2}}{2\tan^{2}\theta }\;;\;p\equiv \eta_{\max}\;\mathrm{and}\;q\propto R_{2}^{2}-R_{1}^{2}.$$

Here, θ is the TF acceptance angle and $\left\{\rho_{1},\rho_{2},\Delta \rho_{1},\Delta \rho_{2}\right\}$ correspond to $\left\{R_{1},R_{2},r_{R1},r_{R2}\right\}$, respectively (see Figure 4). This reduces the complexity from several variables in Equation (2) to just *p* and *q*, where *p* reflects the maximum responsivity, $\eta_{\max}$, achieved at infinite distance, while *q* is proportional to the square distance distance between the two RF collections.

### 3.2. Toy Model Outcomes

**Figure 6:**
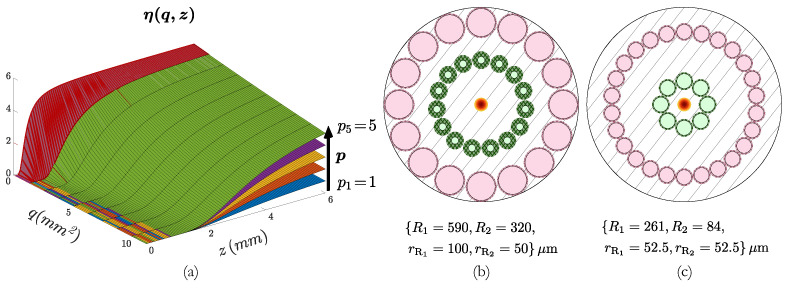
(**a**) Evolution of the toy model OFDS’s responsivity $\eta \left(z\right)$ when varying $q=\left[0,10\right]\;{\mathrm{mm}}^{2}$ every ${\mathrm{mm}}^{2}$ and p=[1,5]. In red, the model $\eta \left(z\right)$ with smallest working range. (**b**) Bundle design with $p=16,q=25.5\;{\mathrm{mm}}^{2}$; (**c**) design for $p=1,q=24.5\;{\mathrm{mm}}^{2}$.

Next, we rendered some models to see the influence of *p* and *q* in the OFDS responsivity, i.e., the behavior of $\eta \left(z\right)$ when rearranging and changing the RFs of the OFDS bundle. The results are shown in Figure 6a. Figure 6b,c show two example bundles corresponding to different {p,q} pairs. The first corresponds to a bundle design with $p=16,q=25.5\;{\mathrm{mm}}^{2}$, and the second was modeled using $p=1,q=24.5\;{\mathrm{mm}}^{2}$.

Figure 6a reveals that low *q* values—red—are associated with OFDSs with a narrower dead zone and higher sensitivity, but a smaller working range. Hence, to improve the working range, we must increase *q*, at the cost of widening the dead zone and losing sensitivity.

### 3.3. Relationship Between the Real Case Geometry and the OFDS Response

**Figure 7:**
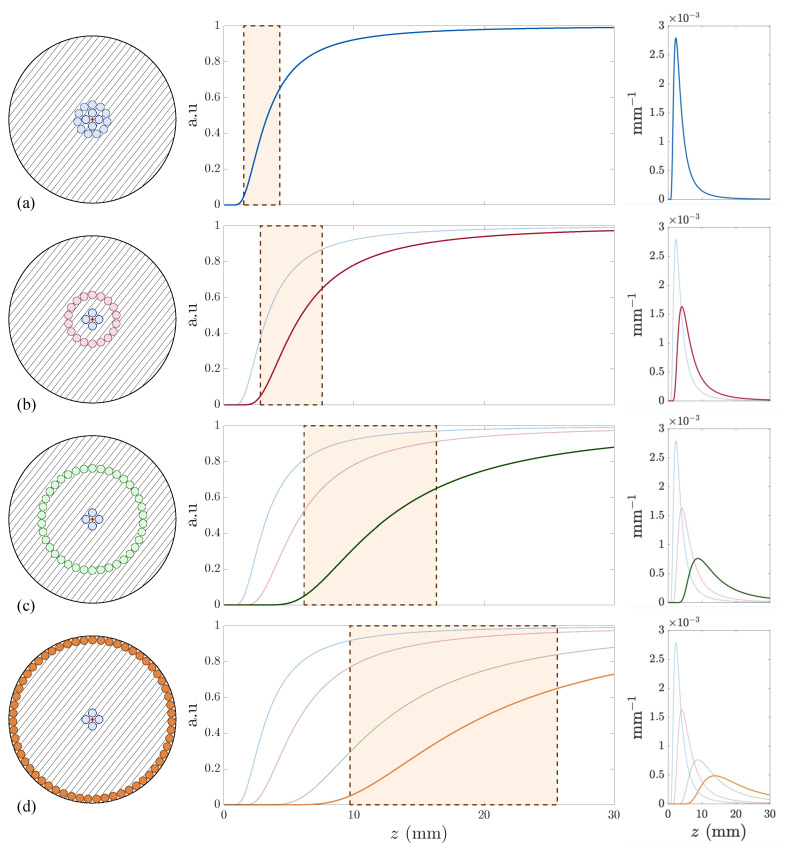
The evolution of the modeled OFDS responsivities. (**Left**): Bundle tips. (**Center**): Responsivities with the linear region in orange. (**Right**): Sensitivities. The 1st RF collection is attached to the TF coating at $R_{1}=173$ µm, whereas the 2nd varies as follows: (**a**–**d**) $R_{2}=\left[390,640,1340,2090\right]$ µm. The TF radius is $r_{T}=2.15$ µm; the RF radii $r_{R1}=r_{R2}=200$ µm; the bundle radius $R_{B}=2200$ µm; and $\theta =5^{\circ }$.

To continue, we attempted to replicate the toy model outcomes within the real case (see Equation (2)) by creating several physical models varying the inter-RF collection distance $R_{2}-R_{1}$, i.e., modifying the value of *q*. The rendered models are depicted in Figure 7.

Figure 7 confirms that the real case aligns with the toy model predictions. Table 1 highlights that achieving a wide working range (Figure 7d) requires maximizing the separation between the first and second RF collections. However, this compromises sensitivity and enlarges the dead zone, both of which are undesirable.

### 3.4. Tetrafurcated OFDS

**Figure 8:**
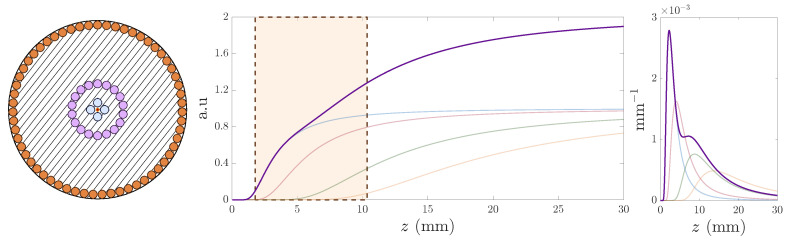
The modeled tetrafurcated OFDS in purple with the previous OFDSs. (**Left**): Bundle tip. (**Center**): Responsivity with the linear region in orange. (**Right**): Sensitivities. The geometrical parameters are in Table 2.

To extend the working range (Figure 7d) while preserving high sensitivity and a small dead zone (Figure 7a), we added another RF collection to the OFDS bundle, resulting in the tetrafurcated OFDS design shown in Figure 8. Its total responsivity is defined as the sum of the responsivities from each RF collection pair.

(5) $$\eta \left(z\right)=\eta_{1}\left(z\right)+\eta_{2}\left(z\right)=\frac{V_{2}+V_{3}}{V_{1}}$$

With the tetrafurcated OFDS design presented in Figure 8, we achieve the metrics outlined in the final entry of Table 1, demonstrating an extended working range and improved sensitivity without notably increasing the dead zone. Moreover, its responsivity nearly doubles compared to previous designs.

## 4. Experimental Results

### 4.1. Experimental Set-Up

**Figure 9:**
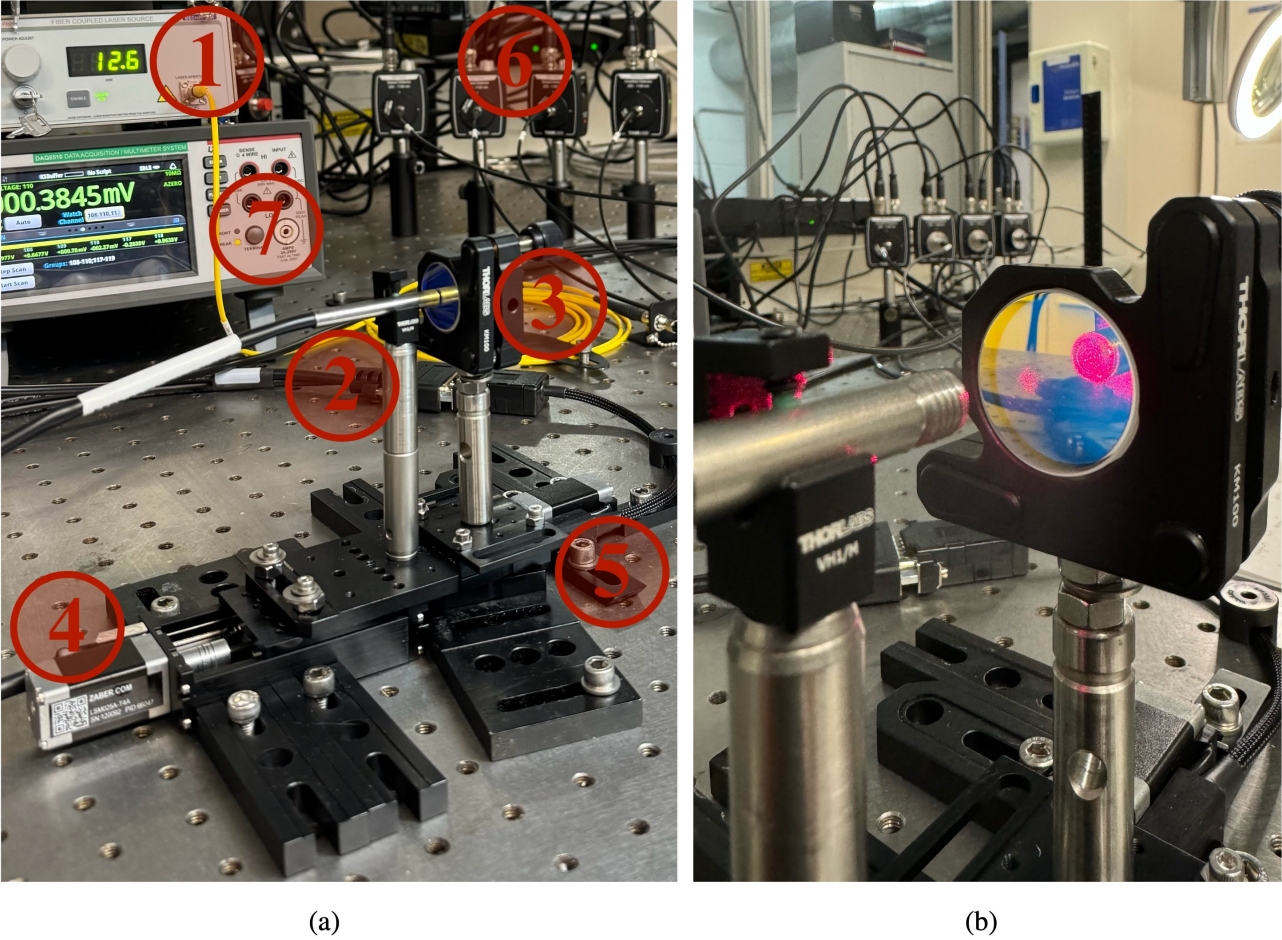
(**a**) Experimental setup for validating the linear displacement theoretical model. Components: (1) 660 nm Fabry–Perot tabletop laser source (S4FC660, Thorlabs, Newton, NJ, USA), (2) OFDS bundle, (3) mirror as a target, (4) linear and (5) angular stages (Zaber, Vancouver, BC, Canada), (6) Thorlabs PDA100A-EC photodetector for each RF collection, and (7) acquisition card (DAQ6510 Keithley, Solon, OH, USA). (**b**) Reflection of the OFDS bundle tip on the mirror.

Figure 1 above presents a schematic diagram of the overall OFDS system, including the laser light source, the fiber bundle, the photodetectors, the acquisition card, and the target. In Figure 9, we show the real-world implementation of that same setup.

In the following, we briefly describe the key aspects of the experiment:

- Laser source and fiber coupling. The laser output at 660 nm is coupled into the TF at the center of the bundle. The reflected light from the target re-enters the bundle through its RF collections and is converted into voltage signals $V_{1}$, $V_{2}$, etc., by the photodetectors.
- Target positioning. The target—here a mirror; in-field, a blade tip—is mounted on a motorized linear stage and/or an angular stage to mimic realistic displacement and orientation changes. This allows us to measure the sensor’s response η(z) over a range of distances and angles.
- Signal processing. Each photodetector output voltage is read by the DAQ6510 acquisition card. Data are recorded at each step of the linear or angular motion. As discussed in Section 3, the ratio $\eta \left(z\right)=V_{2}/V_{1}$—or corresponding sums for higher furcation levels—yields a responsivity curve that reflects the distance measurement capability of the OFDS.

By comparing Figure 1 and Figure 9, one can see how the conceptual layout matches our laboratory configuration.

### 4.2. Tetrafurcated Design Validation

We proved the theoretical model manufacturing a tetrafurcated OFDS with the geometry in Table 2.

**Figure 10:**
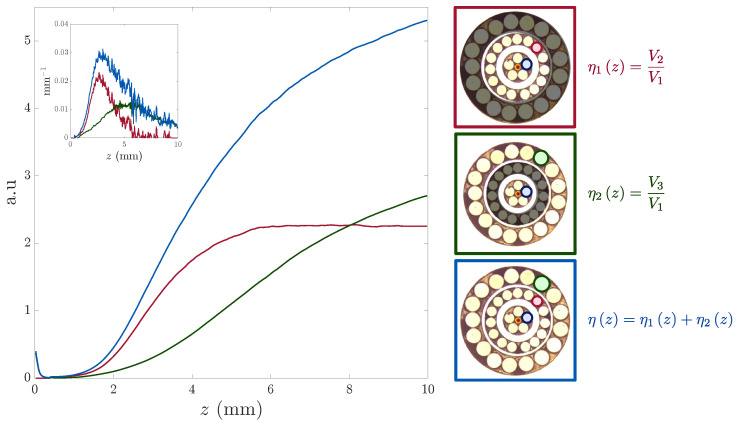
Experimental results of the tetrafurcated OFDS: The red curves represent the responsivities for the 1st and 2nd RF collections, while the green curves correspond to the 1st and 3rd RF collections. The blue curves illustrate the overall OFDS response. The main plot displays the responsivities, with a smaller inset in the upper left corner showing the sensitivities. The corresponding RF collections for each responsivity are indicated on the right.

Figure 10 shows the experimental OFDS responsivity calculated using only the first and second RF collections (red curve). When using the first and third RF collections instead, the responsivity shifts towards greater distances (green curve), indicating a wider dead zone and reduced sensitivity. These results confirm experimentally what we theorized in Section 3.3.

**Figure 11:**
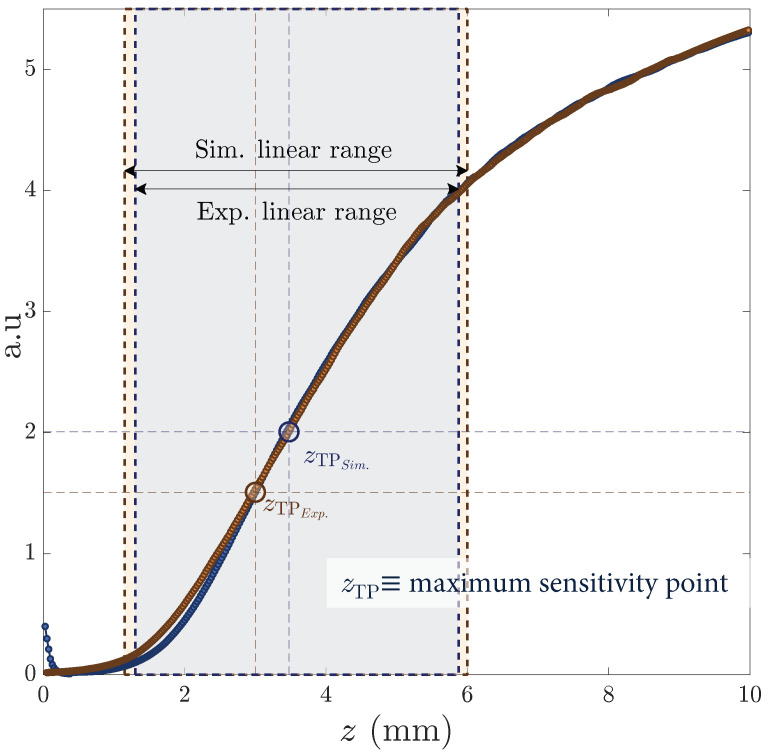
Experimental (blue) and modeled (orange) responsivities of the manufactured tetrafurcated OFDS. The linear regions are highlighted in blue and orange, respectively. $z_{\mathrm{TP}}$ is the turning point of the responsivity, i.e., where the sensitivity is maximum.

Then, we inserted the geometrical specifications of the manufactured tetrafurcated OFDS in our model (see Equations (1), (2) and (5)). Figure 11 presents the comparison between the OFDS responsivity $\eta \left(z\right)$ obtained from the theoretical model and the experimental measurements.

Furthermore, comparing the performance metrics of the theoretical and the manufactured OFDS confirms their strong similarity. The mean square error (MSE) between the theoretical and experimental responsivities (Equation (6)) was 0.26%. This low MSE highlights the accuracy of the theoretical model.

(6) $$\mathrm{MSE}=100\cdot \sum_{i=1}^{N}\frac{{\left(y\left(i\right)-\hat{y\left(i\right)}\right)}^{2}}{N}$$

where *y* and $\hat{y}$ are the modeled and experimental responsivities, respectively. *N* is number of samples, which is the same for both signals. Finally, in Table 2, we provide a side-by-side comparison of the modeled and manufactured OFDSs’ performance. Comparing the trifurcated designs (first two rows) reveals that sensors with a larger inter-RF collection distance, $R_{2}-R_{1}$, achieve a broader working range, while those with closer RF collections exhibit higher sensitivity and a narrower dead zone.

Additionally, comparing the first two designs with the third one, we verify that adding a third RF collection extends the working range and improves sensitivity, all while maintaining a small dead zone. This ensures the sensor retains its capability for accurate measurements at short distances.

### 4.3. Linearized Working Range: Pentafurcated Design

**Figure 12:**
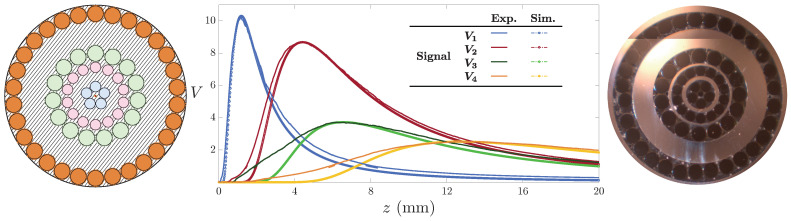
Modeled (dashed, left) and measured (plain, right) OFDS voltage signal for each RF collection with the bundle tips. The geometrical parameters of the OFDS are the following: $\left\{r_{R1},r_{R2}\right\}=100\;\mu m;\left\{r_{R3},r_{R4}\right\}=150\;\mu m;r_{T}=2.15\;\mu m,R_{B}=3050\;\mu m;\;\mathrm{and}\;\theta =5^{\circ }$.

**Figure 13:**
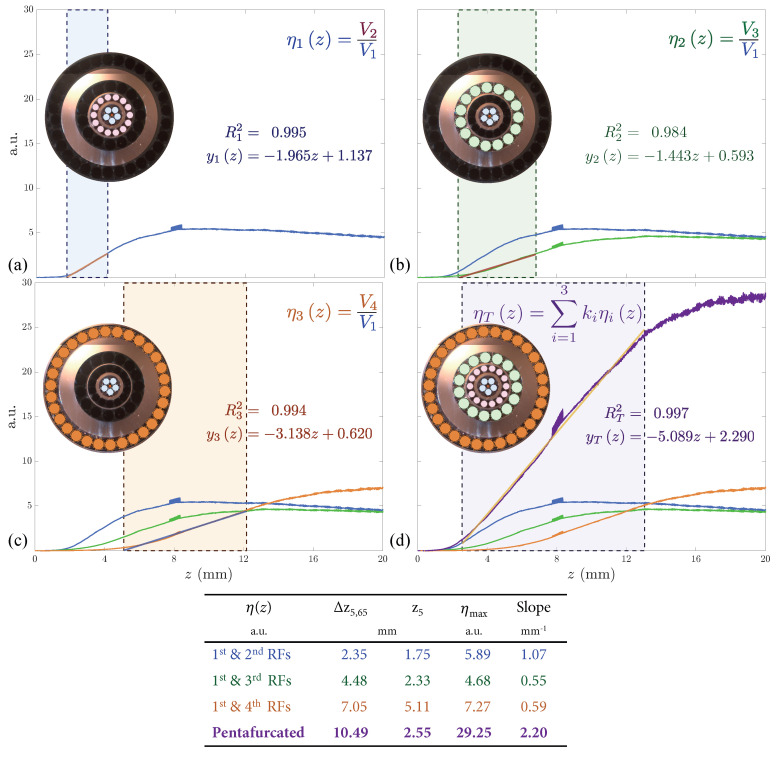
(**a**–**d**) Experimental pentafurcated OFDS responsivity along with the bundle tips. As defined in Equation (2), $k_{i}$ refers to the tunable gain quotient $G_{i}/G_{1}$ of the photodetectors.

Once we had figured out how to extend the working range of the OFDS, we attempted to linearize it, since the responsivity of the tetrafurcated OFDS shown in Figure 8 and Figure 10 appears to have a little irregularity at its center. For that, we developed a final model incorporating a fourth RF collection, i.e., a pentafurcated OFDS. Figure 12 illustrates the modeled and manufactured geometries of the pentafurcated design, along with the individual responses of each RF collection. Both experimental (solid lines) and modeled (dashed lines) responses are displayed, showing strong agreement. This alignment validates the model and highlights the precision of the experimental setup. The achieved responsivity of the pentafurcated OFDS is plotted in Figure 13 in purple, along with the OFDS bundle tip’s geometrical arrangement.

As Figure 13 reveals, a pentafurcated OFDS design does not only extend the working range of the sensor when compared to trifurcated designs, but also completely linearizes it. We first observe the remarkable agreement between the experimental results and the model. In addition to the theoretical design expectations, the experimental results show a significant extension of the linear range from 2.5 mm to 10.49 mm. This range extension makes this bundle suitable for measuring TC at any stage of an aircraft engine.

While this work focuses on tetra- and pentafurcated OFDS designs, we acknowledge the potential benefits and challenges of increasing the number of RF collections beyond five—e.g., 6, 7, or even 10 elements. Adding more RF collections theoretically extends the working range and improves linearity. However, practical limitations must be considered:

- Physical constraints: Increasing the number of elements reduces the space available for each fiber within the bundle. This requires precision manufacturing techniques, raising costs and complexity.
- Photodetector requirements: Each RF collection requires a dedicated photodetector, amplifying the cost of the system, its size, and its complexity.
- Bundle size limitations: Aeronautical applications impose strict constraints on the physical size of the sensor due to the limited space available in jet-engine stages.

In summary, while designs with more elements may offer wider working ranges and improved performance, practical considerations such as manufacturing complexity, system cost, and space constraints impose an effective limit.

Finally, in Table 3, we have compared our pentafurcated OFDS performance with other similar OFDSs. The proposed displacement sensor has not only the widest sensing range, but also a high sensitivity, while maintaining low cost, small size, and easy fabrication, which are required to monitor aircraft engines [25].

Mechanical vibrations, such as those encountered in turbine systems, are indeed a critical factor in real-world applications. We have already demonstrated that optical fiber displacement sensors can effectively measure blade vibrations in tip timing applications, as shown in [22]. Their OFDS design successfully captured vibrational data under operational conditions.

Given that our design significantly enhances sensitivity and extends the working range, we anticipate even greater precision in measuring dynamic displacements, including blade vibrations. These improvements should result in more accurate tracking of vibrational modes, enabling a more detailed analysis of structural integrity in aeronautical systems, including early-stage crack detection.

## 5. Conclusions

In this research, we have demonstrated, both theoretically and experimentally, the relationship between the geometric configuration of an OFDS (fiber placement and radii) and the linear range of its response using the toy and Gaussian model approaches.

We have designed and manufactured tetra- and pentafurcated OFDSs that significantly enhance the working range (10.49 mm) and sensitivity (2.20 mm^−1^) compared to previous designs, while maintaining a small dead zone (2.5 mm).

These advancements represent a step forward in OFDS sensor development, enabling a single bundle to serve multiple applications (TC and TT), across various engine stages.

We also have experimentally validated the theoretical response of the both OFDSs. The physical modelization and measurements are in good agreement with an MSE of 0.26%.

Finally, the pentafurcated OFDS emerges as an optimal solution for aeronautical applications, offering an exceptional balance between an extended working range, sensitivity, and the complexity of the setup.

## Figures and Tables

**Table 1:** Summary of modeled OFDSs and the extracted points of interest (PoIs).

OFDS Type	Model	$R_{1}$	$R_{2}$	$R_{3}$	$\Delta z_{5,65}$	$z_{5}$	$\eta_{\max}$	$\Delta z_{5,65}$ Slope
		(mm)			(mm)	(mm)	(a.u.)	(mm^−1^)
Trifurcated	a	0.17	0.39	−	22.76	1.54	0.99	0.22
	b	0.17	0.64	−	24.75	2.81	0.98	0.13
	c	0.17	1.34	−	10.18	6.17	0.94	0.05
	d	0.17	2.09	−	15.94	9.70	0.86	0.03
Tetrafurcated	-	0.17	1.13	2.09	28.57	1.78	1.97	0.14

$R_{B}=1100\;\mu m;r_{T}=2.15\;\mu m;\left\{r_{R1},r_{R2},r_{R3}\right\}=100\;\mu m;\;\mathrm{and}\;\theta =5^{\circ }$.

**Table 2:** Summary of modeled OFDSs and the extracted points of interest (PoIs), along with the geometrical arrangement of the manufactured tetrafurcated fiber bundle.

	OFDS	RF Collections	$\Delta z_{5,65}$	$z_{5}$	$\eta_{\max}$	$\Delta z_{5,65}$ Slope
			(mm)		(a.u.)	(mm^−1^)
Experimental	Trifurcated	1st & 2nd	2.20	1.3	2.27	0.62
		1st & 3rd	4.47	2.0	2.70	0.36
	Tetrafurcated	1st & 2nd + 1st & 3rd	3.53	1.5	5.30	1.50
Modelization	Tetrafurcated	1st & 2nd + 1st & 3rd	3.77	1.2	5.27	1.43

$R_{B}=1120\;\mu m;r_{T}=2.15\;\mu m;\left\{r_{R1},r_{R2}\right\}=100\;\mu m;\left\{r_{R3},r_{R4}\right\}=170\;\mu m;,\;\mathrm{and}\;\theta =5^{\circ }$.

**Table 3:** Performance comparison of OFDSs in the literature, sorted by linear range.

Performance Comparison			
**Principle**	**Range (mm)**	**Sensitivity (${\mathrm{mm}}^{-1}$)**	**Ref.**
Intensity-based	10.73	4.28	[32]
	10.90	1.77	[33]
	10.50	0.86	[34]
	12.80	0.32	[35]
	12.80	0.13	[36]
	13.00	0.33	[37]
	15.00	0.50	[38]
	16.00	0.75	[39]
	16.90	0.41	[27]
	10.49	2.20	Our OFDS

**Figure A1:**
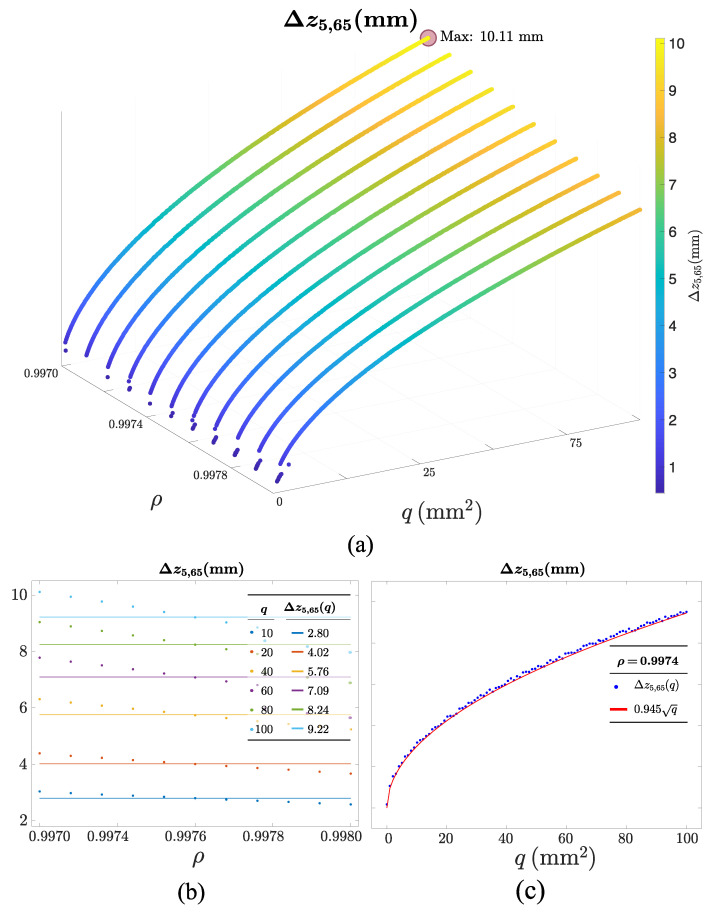
(**a**) Relationship between the linear range, $\Delta z_{5,65}$, the Pearson’s correlation coefficient ρ, and the parameter *q*. The data correspond to a toy model trifurcated design. The linear range increases with the square root of *q* and decreases linearly with ρ. (**b**) Linear range $\Delta z_{5,65}$ as a function of ρ. The lines represents the linear range for different values of *q*. (**c**) Comparison of linear range definitions. The linear range values for ρ=0.9974 (blue) and $\Delta z_{5,65}$ (red) are plotted against *q*. This comparison validates their computational interchangeability.

## Data Availability

The original contributions presented in the study are included in the article; further inquiries can be directed to the corresponding author.

## Appendix A. Definitions of the Linear Range

In this appendix, we justify the equivalence between the two definitions of the linear range of our sensor. As we will observe, we can use both definitions interchangeably, as they are equivalent and lead to the same results.

Figure A1a shows that for a fixed ρ, the linear range increases with the square root of *q*, meaning it mainly depends on the distance between the RFs collections; it is also observed that the linear range decreases linearly with ρ. Figure A1b further illustrates $\Delta z_{5,65}$ as a function of ρ (points) for various *q* values (dashed lines). The data correspond to a trifurcated design.

Both values of the linear range, Pearson’s and $\Delta z_{5,65}$, coincide for a Pearson’s correlation coefficient value between [0.9972, 0.9974], independently of the *q* value, ranging from q=[10,100]). In Figure A1c, the linear range is defined by a Pearson’s coefficient value of 0.9974 (blue) and $\Delta z_{5,65}$ (red) for different values of *q*.

It is observed that both definitions are indistinguishable, although $\Delta z_{5,65}$ is much less costly from a computational standpoint.
